# Nomogram-derived prediction of pathologic complete response (pCR) in breast cancer patients treated with neoadjuvant chemotherapy (NCT)

**DOI:** 10.1186/s12885-020-07621-7

**Published:** 2020-11-19

**Authors:** Shengyu Pu, Ke Wang, Yang Liu, Xiaoqin Liao, Heyan Chen, Jianjun He, Jian Zhang

**Affiliations:** Present Address: Department of Breast Surgery, the First Affiliated Hospital, Xi’an Jiaotong University, 277 Yanta Western Rd., Xi’an, 710061 Shaan’xi Province China

**Keywords:** Breast cancer, Neoadjuvant chemotherapy, Nomogram, pCR

## Abstract

**Background:**

Previous research results on the predictive factors of neoadjuvant chemotherapy (NCT) efficacy in breast cancer are inconsistent, suggesting that the ability of a single factor to predict efficacy is insufficient. Combining multiple potential efficacy-related factors to build a model may improve the accuracy of prediction. This study intends to explore the clinical and biological factors in breast cancer patients receiving NCT and to establish a nomogram that can predict the pathologic complete response (pCR) rate of NCT.

**Methods:**

We selected 165 breast cancer patients receiving NCT from July 2017 to May 2019. Using pretreatment biopsy materials, immunohistochemical studies to assess estrogen receptor (ER), progesterone receptor (PR), human epidermal growth factor receptor 2 (HER-2), and Ki-67 expression. The correlation between biological markers and pCR was analyzed. These predictors were used to develop a binary logistic regression model with cross-validation and to show the established predictive model with a nomogram.

**Results:**

The nomogram for pCR based on lymphovascular invasion, anemia (hemoglobin≤120 g/L), ER, Ki67 expression levels and NCT regimen had good discrimination performance (area under the curve [AUC], 0.758; 95% confidence interval [CI], 0.675–0.841) and calibration coordination. According to the Hosmer-Lemeshow test, the calibration chart showed satisfactory agreement between the predicted and observed probabilities. The final prediction accuracy of cross-validation was 76%.

**Conclusions:**

We developed a nomogram based on multiple clinical and biological covariations that can provide an early prediction of NCT response and can help to quickly assess the individual benefits of NCT.

**Supplementary Information:**

The online version contains supplementary material available at 10.1186/s12885-020-07621-7.

## Background

Breast cancer is the most common malignant tumor among women and the second leading cause of cancer-related death in America. There has been a gradual increase in the incidence of breast cancer [1]. Neoadjuvant chemotherapy (NCT), which is increasingly offered to breast cancer patients, is mainly used to reduce tumor burden to enable breast-conserving surgery (BCS) and provide the opportunity to assess the response to treatment by an in vivo chemosensitivity test [2–5]. “Patient level analysis” has shown that achieving pathologic complete response (pCR) under NCT is associated with an increase in event-free survival (EFS) and overall survival (OS) [4, 6–8]. Several studies have found that BC patients who achieve pCR after NCT exhibit improved survival [6, 9]. However, only 5–38% of BC patients achieve pCR, meaning that the majority (those who cannot attain pCR) have a higher risk of death and recurrence [4, 8]. Some baseline clinicopathological features can predict the curative effect of NCT for breast cancer and further reflect the long-term outcomes of patient treatment. These factors should help us select the patients who will benefit most from treatment and recommend a tailored approach when choosing the initial treatment to give patients the best response to treatment and subsequent overall survival [9, 10].

Nomograms in clinical settings are considered to be comprehensive predictive tools that can estimate the probabilities, risks or clinical outcomes. There have been few well-designed nomograms predicting the probability of pCR in the literature, and the implications of providing a detailed probability of pCR in patients who receive NCT have not been well established. For those reasons, based on preoperative clinicopathological variables and simple laboratory indexes, we established a nomogram to calculate the probability of pCR in breast cancer patients who received NCT.

## Methods

### Study population

This study included 165 invasive breast cancer patients who receiving 2–6 cycles of NCT before surgery from July 2017 to May 2019. The chemotherapy regimens consisted of docetaxel + epirubicin + cyclophosphamide (TEC) and docetaxel + carboplatin + trastuzumab (TCbH) every 3 weeks before surgery [11]. Suspended treatment when serious chemotherapy toxicity, disease progression, or other diseases unsuitable for chemotherapy occurred. Surgical treatment of the breast and axilla lymph nodes was performed within 1 month after the completion of NCT. Combining the clinical evaluations before NCT and postoperative pathology reports, we determined whether further therapy was needed after surgery. Specific details of this process were described in our previous studies [12].

The exclusion criteria have been described in our previous study [12], which mainly included: 1. stage IV breast cancer; 2. luminal A breast cancer (luminal A subtypes exhibit lower sensitivity to chemotherapy, and hormonal therapy alone is the preferred treatment for this subtype [13]); 3. male breast cancer; 4. other types of neoadjuvant therapy, including radiotherapy and endocrine therapy. Finally, a total of 165 breast cancer patients who received NCT were included.

This research was a retrospective study without other diagnostic or therapeutic measures; therefore, informed consent was waived. But our research was approved by the Medical Ethics Review Committee of the First Affiliated Hospital of Xian jiaotong University.

### Data collection

We extracted the following data from the patient’s medical records: sex, BMI, menopausal status, tumor multifocality, size and stage, local invasion, lymphovascular invasion, histological type and grade, and Ki67, estrogen receptor (ER), progesterone receptor (PR), human epidermal growth factor receptor 2 (HER-2), hemoglobin levels and NCT regimen, time and pathological outcomes. The clinical tumor size was assessed by ultrasound (categorized as T1 ≤ 2 cm, 2 cm < T2 ≤ 5 cm, and T3 > 5 cm) and lymph node status was assessed by CNB when the physical examination and ultrasound showed lymph node was positive (categorized as N0 and N1–2). The local invasion was defined as invasion of the chest wall and skin. The multifocality of tumors was categorized as multifocal or unifocal, and pathological types included invasive ductal carcinoma (IDC) and special type of breast cancer such as invasive lobular carcinoma, metaplastic carcinoma and medullary carcinoma. Finally, dedicated breast pathologists performed the diagnostic biopsy and evaluation of excised specimens. The expression of ER, PR, HER2 and Ki67 was evaluated by immunohistochemical (IHC) staining, and the level of ER and PR less than 1% was considered negative, the absence of both ER and PR expression was defined as HR negative, and the presence of either was defined as HR positive [14]. HER2 positivity was defined as 3+ according to IHC analysis or amplification confirmed by FISH; scores 0 or 1 + were defined as HER2 negative [15]. Markers of LVI included D2–40 for lymphatic endothelium and CD31 for all vascular endothelium. 1000 infiltrating cancer cells with a representative area were selected for counting Ki67 after evaluating the entire section, not less than 500 cancer cells. Finally, the Ki67 expression was divided into two groups according to the optimal cutoff values determined by maximizing the Youden index (sensitivity + specificity - 1) using receiver operating characteristic (ROC) curve analyses: Ki67 > 60% and Ki67 ≤ 60%. The molecular subtypes included three categories: luminal B subtype (ER+ and/or PR+, any HER2 status, Ki67 > 30%), HER-2 enriched subtype (ER-, PR-, HER2+), and triple-negative subtype (ER-, PR-, HER2-) [16]. Evaluation of the pathological response after NCT was based on the Miller/Payne grading system (MP): pCR was defined as an absence of residual invasive carcinoma in the breast (postoperative MP1–4 was defined as non-pCR; MP5 was defined as pCR) [17].

### Statistical analysis

We used univariate and multivariate binary logistic regression analyses to assess the effect of target variables on pCR status. Combined with clinical and statistical significance, we screened for potential predictors of efficacy and established a logistic regression model. The model was then used to make a nomogram and predict the probabilities of each patient achieving pCR from NCT. After establishing the model, the predictive ability of the model was evaluated by discrimination and correction. The degree of discrimination was measured by the receiver operating characteristic curve (ROC curve) area under the curve (AUC). The correction was mainly evaluated by the calibration curve. We used the 5-fold cross-validation method to evaluate the discrimination ability to obtain a relatively unbiased estimate and used the Hosmer-Lemeshow goodness of fit test to assess the calibration curve. The diagnostic odds ratio was calculated to further evaluate the performance of the nomogram. The range of diagnostic ratio was a minimum value of 0 and a maximum value of infinity, with a higher value combined with a better performance on the discrimination test. A value of 1 indicated that the test could not distinguish patients with the disease from patients without the disease. Statistical analyses were performed using IBM SPSS Statistics 24.0 software (IBM Corporation, Armonk, NY, USA) and R version 3.3.3 software (The R Foundation for Statistical Computing, Austria, Vienna). A *P* value≤0.05 was deemed statistically significant.

## Results

### Patient characteristics

**Fig. 1:**
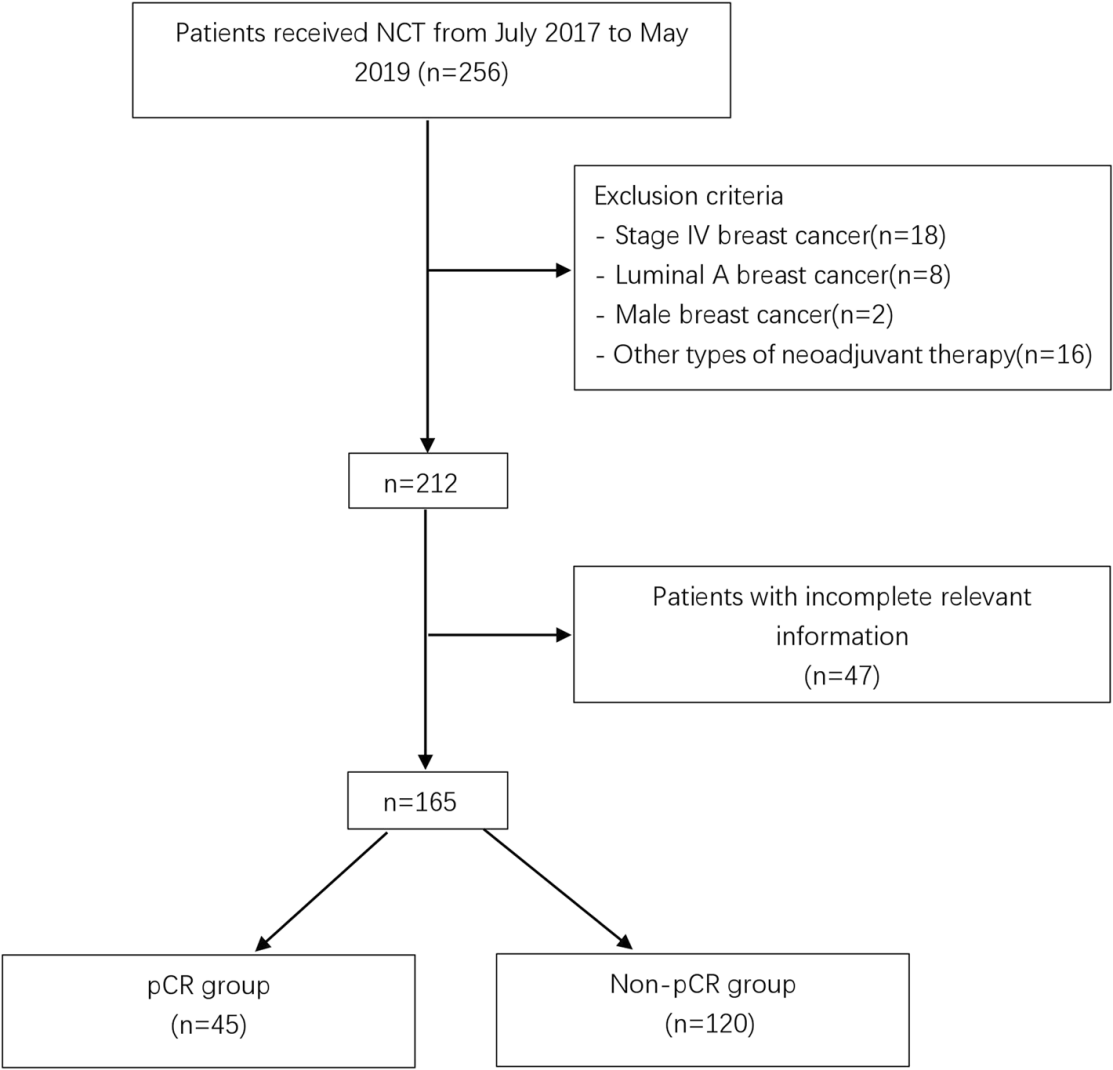
Flow diagram of selection method

In this study, we retrospectively collected a total of 165 patients with operable breast cancer and summarized their clinical characteristics in Table 1. The details of the patient selection process are summarized in Fig. 1. Of the 165 patients, 45 patients (27.3%) showed pCR after receiving NCT. The median age was 45 years, 44 (26.7%) patients had a BMI ≥ 25, 88 (53.33%) patients were premenopausal, 37 (22.4%) patients had multiple foci of carcinoma, 7 patients (4.24%) had local invasion, and 41 patients (24.8%) developed lymphovascular invasion. Based on the 8th TNM staging system recommended by the AJCC, among 165 patients, before NCT, 11.5% (19 patients) were classified with cT1, 74.5% (123 patients) were classified with cT2, 13.9% (23 patients) were classified with cT3, 31.5% (52 patients) were classified with cN0, and 68.5% (113 patients) were classified with cN1–2. There were 53 patients with histological grade I-II and 112 patients with histological grade III tumors. Additionally, 152 (92.1%) patients were diagnosed with invasive ductal cancer, and 13 (7.9%) patients were diagnosed with other types of breast cancer. The positive expression rate of ER in the non-pCR group was 39.2%, and it was 15.6% in the pCR group. The positive PR expression rates in the non-pCR group and pCR group were 55.0 and 35.6%, respectively. HER2 status was positive in 81 patients (49.1%) and negative in 84 patients. Ki67 expression was high (> 60%) in 47 patients and low (≤60%) in 118 patients. Eighty-six patients received NCT regimens containing TEC, and 79 patients received NCT regimens containing TCbH. The percentage of luminal B cancer in the pCR group was 4.4%, and that in the non-pCR group was 35.8%. The percentage of HER2-enriched tumors in the pCR group and non-pCR group was 60.0 and 35.0%, respectively, and that of triple-negative tumors in the pCR group and non-pCR group was 35.6 and 29.2%, respectively.

**Table 1:** Clinicopathological characteristics of 165 patients treated with neoadjuvant chemotherapy (NCT)

Factors	No. (%)		
	Total	pCR	non-pCR
**age (years)**			
≤ 45	63 (38.18)	19 (42.22)	44 (36.67)
> 45	102 (61.82)	26 (57.78)	76 (63.33)
**BMI (kg/m**^**2**^**)**			
< 25	121 (73.33)	35 (77.78)	86 (71.67)
≥ 25	44 (26.67)	10 (22.22)	34 (28.33)
**Menopausal status**			
Postmenopausal	77 (46.67)	22 (48.89)	55 (45.83)
Premenopausal	88 (53.33)	23 (51.11)	65 (54.17)
**Multifocality**			
Multifocal	37 (22.42)	9 (20.00)	28 (23.33)
Unifocal	128 (77.58)	36 (80.00)	92 (76.67)
**Local invasion**			
Yes	7 (4.24)	1 (2.22)	6 (5.00)
No	158 (95.76)	44 (97.78)	114 (95.00)
**LVI**			
Absent	124 (75.15)	40 (88.89)	84 (70.00)
Present	41 (24.85)	5 (11.11)	36 (30.00)
**Clinical tumor size**			
T1	19 (11.52)	6 (13.33)	13 (10.83)
T2	123 (74.55)	32 (71.11)	91 (75.83)
T3	23 (13.94)	7 (15.56)	16 (13.33)
**Lymph node status**			
N0	52 (31.52)	13 (28.89)	39 (32.50)
N1–2	113 (68.48)	32 (71.11)	81 (67.50)
**Histological grade**			
I-II	53 (32.12)	12 (26.67)	41 (34.17)
III	112 (67.88)	33 (73.33)	79 (65.83)
**Histologic type**			
IDC	152 (92.12)	44 (97.78)	108 (90.00)
Others	13 (7.88)	1 (2.22)	12 (10.00)
**Estrogen receptor**			
Negative	111 (67.27)	38 (84.44)	73 (60.83)
Positive	54 (32.73)	7 (15.56)	47 (39.17)
**Progesterone receptor**			
Negative	83 (50.30)	29 (64.44)	54 (45.00)
Positive	82 (49.70)	16 (35.56)	66 (55.00)
**Hormone receptor**			
Negative	82 (49.70)	28 (62.22)	54 (45.00)
Positive	83 (50.30)	17 (37.78)	66 (55.00)
**HER2**			
Negative	84 (50.91)	18 (40.00)	66 (55.00)
Positive	81 (49.09)	27 (60.00)	54 (45.00)
**Ki67**			
≤ 60%	118 (71.52)	25 (55.56)	93 (77.50)
> 60%	47 (28.48)	20 (44.44)	27 (22.50)
**Molecular subtype**			
Luminal B	45 (27.27)	2 (4.44)	43 (35.83)
HER2 enriched	69 (41.82)	27 (60.00)	42 (35.00)
Triple negative	51 (30.91)	16 (35.56)	35 (29.17)
**NCT regimen**			
TEC	86 (52.12)	17 (37.78)	69 (57.50)
TCbH	79 (47.88)	28 (62.22)	51 (42.50)
**NCT time, cycles**			
≤ 4	28 (16.97)	4 (8.89)	24 (20.00)
> 4	137 (83.03)	41 (91.11)	96 (80.00)
**Hemoglobin(g/L)**			
≤ 120	42 (25.45)	6 (13.33)	36 (30.00)
> 120	123 (74.55)	39 (86.67)	84 (70.00)

Abbreviations: *NCT* Neoadjuvant chemotherapy, *BMI* body mass index, *IDC* Invasive ductal carcinoma, *HER2* Human epidermal growth factor receptor 2, *LVI* lymphovascular invasion, Others: invasive lobular carcinoma, metaplastic carcinoma and medullary carcinoma, *pCR* pathologic complete response

### Predictors of pCR

Table 2 shows the results of univariate and multivariate analyses. There was a significant correlation between pCR and lymphovascular invasion (*P* = 0.017), ER (*P* = 0.028), PR (*P* = 0.006), molecular subtype, Ki67 (*P* = 0.006), NCT regimen (*P* = 0.025) and anemia (hemoglobin≤120 g/L) (*P* = 0.033). Independent predictors were determined in the multivariate logistic regression analysis (*P* < 0.05), and the NCT regimen, ER, anemia, lymphovascular invasion and Ki67 were utilized to construct the nomogram. Patients with lymphovascular invasion were associated with a lower pCR rate (odds ratio (OR) 0.314, 95% CI 0.108–0.918, *P* = 0.034). Anemia was also associated with pCR. There was a low pCR rate when hemoglobin levels were ≤ 120 g/L (OR 0.423, 95% CI 0.154–0.988, *P* = 0.046). Negative ER was associated with higher pCR rates (OR 0.342, 95% CI 0.132–0.889, *P* = 0.028), and Ki67 > 60% was also associated with higher pCR rates (OR 0.258, 95% CI 0.106–0.627, *P* = 0.003). Additionally, patients with TCbH were more likely to achieve pCR than those with TEC (OR 3.268, 95% CI 1.380–7.738, *P* = 0.007).

**Table 2:** Univariate and multivariate analysis of clinical and pathologicalal response rates by biological factors

Factors	Univariate analysis			Multivariate analysis		
	OR	95%CI	***P-***value	OR	95%CI	***P***-value
**age (years)**						
≤ 45	Ref					
> 45	0.792	0.394–1.593	0.513			
**BMI (kg/m**^**2**^**)**						
< 25	Ref					
≥ 25	1.384	0.617–3.102	0.430			
**Menopausal status**						
Postmenopausal	Ref					
Premenopausal	1.130	0.569–2.245	0.726			
**Multifocality**						
Multifocal	Ref					
Unifocal	0.821	0.353–1.911	0.648			
**Local invasion**						
Yes	Ref					
No	0.432	0.051–3.690	0.443			
**LVI**						
Absent	Ref					
Present	0.292	0.106–0.800	0.017	0.314	0.108–0.918	0.034
**Clinical tumor size**						
T1	Ref					
T2	0.762	0.267–2.173	0.533			
T3	0.948	0.255–3.525	0.875			
**Lymph node status**						
N0	Ref					
N1–2	1.185	0.560–2.507	0.657			
**Histological grade**						
I-II	Ref					
III	1.427	0.667–3.054	0.360			
**Histologic type**						
IDC	Ref.		.			
Others	4.889	0.617–38.739	0.133			
**Progesterone receptor**						
Negative	Ref.		.			
Positive	0.451	0.222–0.917	0.028			
**Estrogen receptor**						
Negative	Ref			Ref		
Positive	0.286	0.118–0.694	0.006	0.342	0.132–0.889	0.028
**Hormone receptor**						
Negative	Ref					
Positive	0.497	0.246–1.002	0.051			
**HER2**						
Negative	Ref					
Positive	1.833	0.914–3.678	0.088			
**Ki67**						
≤ 60%	Ref.		.	Ref		
> 60%	0.363	0.175–0.751	0.006	0.258	0.106–0.627	0.003
**Molecular subtype**						
Luminal B	Ref.		.			
HER2 enriched	0.072	0.016–0.324	0.001			
Triple negative	0.711	0.331–1.527	0.046			
**NCT regimen**						
TEC	Ref			Ref		
TCbH	2.228	1.103–4.501	0.025	3.268	1.380–7.738	0.007
**NCT time, cycles**						
≤ 4	Ref.		.			
> 4	0.390	0.127–1.196	0.100			
**Hemoglobin(g/L)**						
≤ 120	Ref					
> 120	0.359	0.140–0.923	0.033	0.423	0.154–0.988	0.046

Abbreviations: *OR* odds ratio, *CI* confidence interval, *BMI* body mass index, *pCR* pathologic complete response, *NCT* Neoadjuvant chemotherapy, *IDC* Invasive ductal carcinoma, *HER2* Human epidermal growth factor receptor, *LVI* lymphovascular invasion, Others: invasive lobular carcinoma, metaplastic carcinoma and medullary carcinoma

### Development and validation of the Nomogram for pCR

**Fig. 2:**
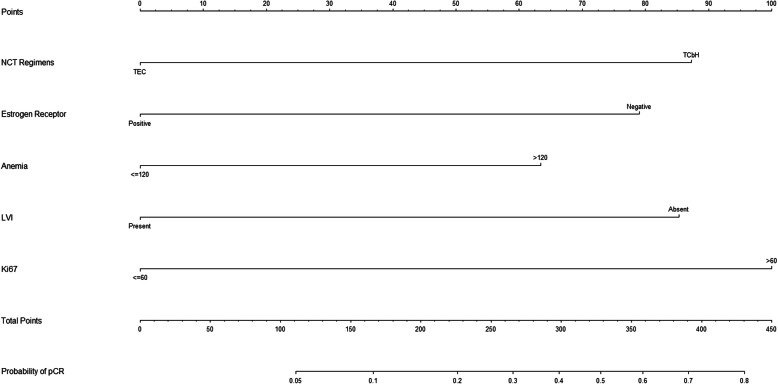
Nomogram to predict the probability of the pathologic complete response (pCR) Abbreviations: NCT: Neoadjuvant chemotherapy; LVI: lymphovascular invasion.

**Fig. 3:**
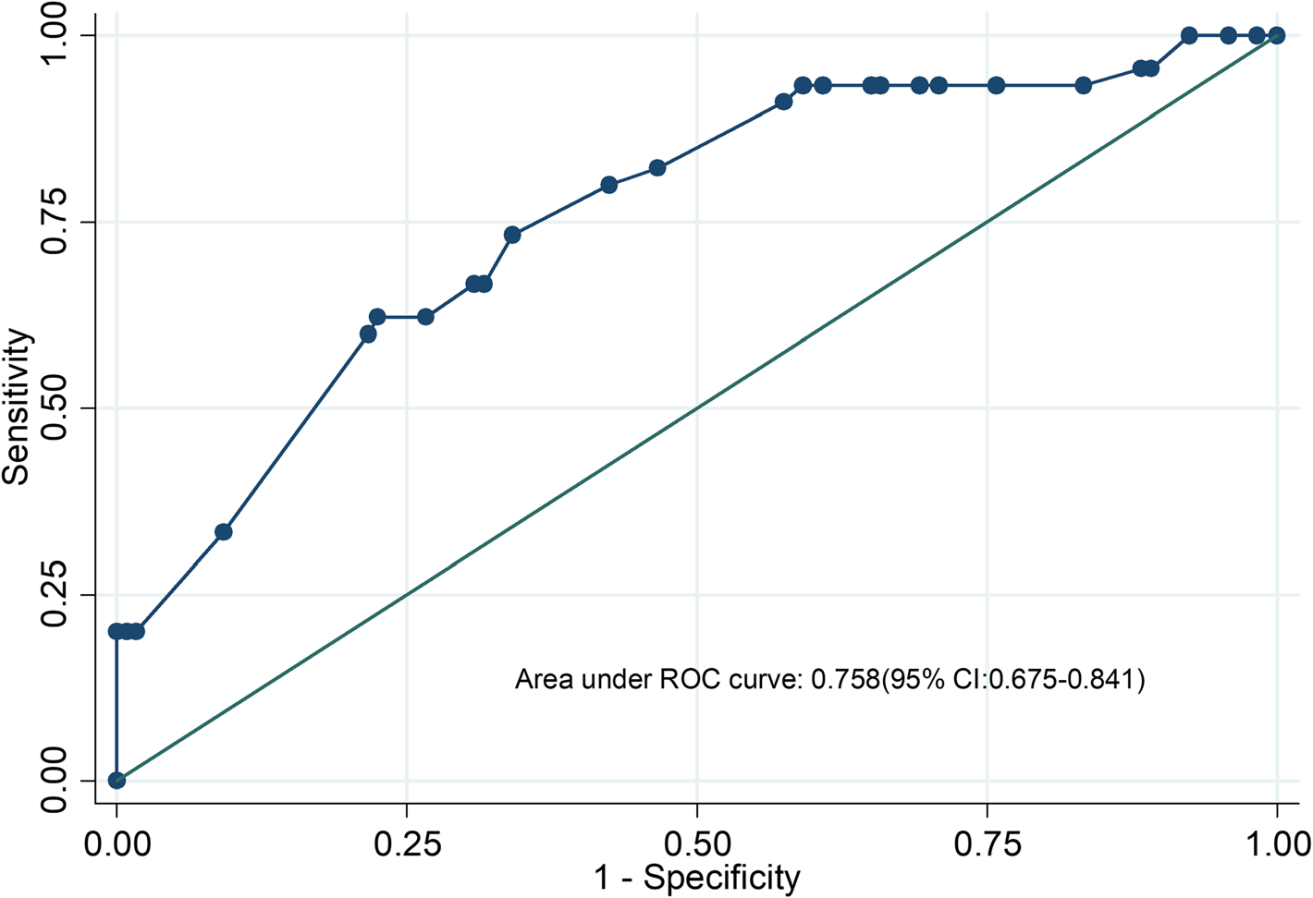
The receiver-operating characteristics (ROC) curve for validating nomogram model. (The AUC was 0.758 (95% CI = 0.675–0.841))

**Fig. 4:**
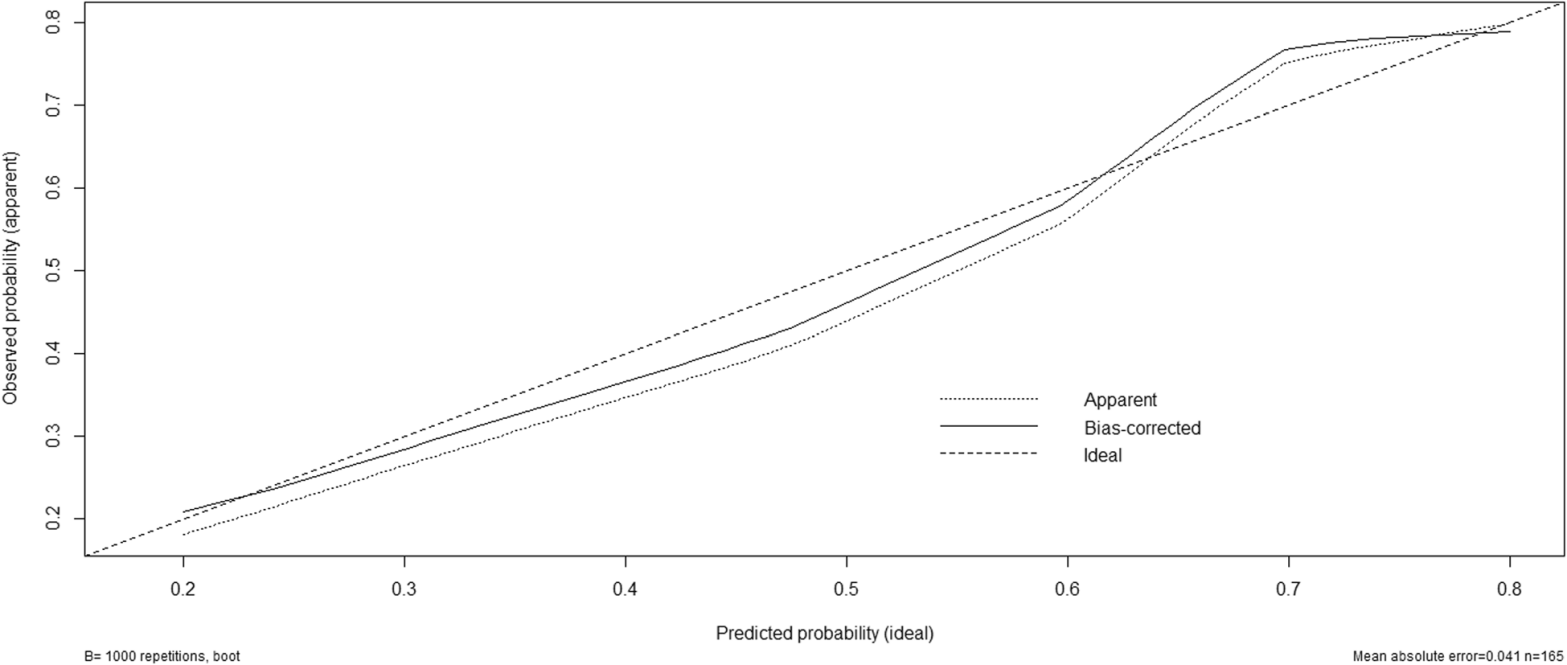
The calibration plot for probability of the pCR nomogram construction

A nomogram was developed based on lymphovascular invasion, anemia, ER expression level, Ki67 expression level, and NCT regimen to predict the pCR rate of NCT among patients (Fig. 2). To calculate the probability of pCR, the scores of five factors were summarized, and the total scores and the bottom risk scale were referenced (Supplementary Table 1). The ROC curve of the nomogram is shown in Fig. 3; the AUC was 0.758 (95% CI = 0.675–0.841). The calibration chart showed good and satisfactory agreement between the predicted probability and the observed probability according to an administered Hosmer-Lemeshow test (Fig. 4) (Hosmer-Lemeshow test, *X*^2^ = 2.986, *P* = 0.965). We performed a 5-fold crossvalidation model with a random split analysis in a cohort of patients, and the final prediction accuracy was 76%.

### Determining the cutoff value for predicting pCR after NCT

The values of sensitivity and specificity and the predictive values of the predicted probability under different cutoff values of the nomogram are shown in Supplementary Table 2. A lower cutoff value resulted in a higher sensitivity and negative predictive value, and the lower specificity and positive predictive value increased. The diagnostic odds ratios of the nomogram at different cutoff values are shown in Supplementary Table 3. The cutoff values for good performance of the nomogram ranged between ≥0.1 and ≥ 0.5. Finally, the optimal cutoff value determined by Youden’s method [18] was 0.32 (sensitivity: 62.2%, specificity: 77.5%, positive predictive value: 50.9%, negative predictive value: 84.5%) (Supplementary Table 4).

### Prospective applications of the Nomogram

To show the application of the nomogram, we took two pathologically proven breast cancer patients who had received NCT as examples. Patient 1 was a 42-year-old female with hollow needle puncture and confirmed grade III invasive ductal carcinoma of the right breast. Routine blood tests showed a hemoglobin level of 132 g/L (63), and IHC suggested ER (−) (79), PR (−), and HER2 (2+). FISH suggested gene amplification, Ki67 (+ 70%) (100), D2–40 (−), no lymphovascular invasion (85), 6-cycle TCbH regimen (87), and a final score of 414. The rate of pCR after NCT was 0.7–0.8, which was greater than 0.32. Therefore, there was a high probability (0.7–0.8) that she would achieve pCR after NCT, and her actual status after NCT was pCR. Patient 2 was a 58-year-old female. Hollow needle puncture confirmed grade II invasive ductal carcinoma of the left breast. Routine blood tests showed a hemoglobin level of 108 g/L (0). IHC suggested the following: ER (60% +) (0), PR (−), HER2 (−), Ki67 (+ 70%) (100), D2–40 (−), no lymphovascular invasion (85), and 6-cycle TEC protocol (0). Her final score was 185, and the rate of pCR after NCT was 0.1–0.2, which was less than 0.32. She had a low probability (0.1–0.2) of reaching pCR after NCT, and her actual status after NCT was non-pCR.

## Discussion

Reliable markers of chemosensitivity help select patients who most benefit from NCT. Additionally, the early identification of nonresponsive patients will protect these patients from unnecessary toxicity caused by ineffective chemotherapy and will provide alternative effective treatment schemes for tumor biological characteristics. Therefore, finding clinical or molecular markers that can predict the efficacy of NCT and then screening patients who can benefit from chemotherapy have been hot research topics in recent years.

However, identifying reliable predictive factors for NCT in breast cancer remains a challenge. In a previous study, not all patients with HER-2-positive disease were treated according to recent guidelines with the standard agent trastuzumab [19]. Hwang HW et al. performed a nomogram to predict the pCR of NCT in breast cancer patients and used a calibration plot to assess the agreement between the predicted and observed probabilities, but they did not use a bootstrap method to validate the model internally or externally [20]. Rouzier et al. performed a nomogram according to clinical characteristics such as tumor size, patient age and hormonal status. However, due to the long history, preoperative chemotherapy regimens have been developed, and this nomogram fails to guide existing clinical treatment [21]. Based on this, researchers are attempting to predict the efficacy of neoadjuvant chemotherapy through a multifactorial digital model. Due to the heterogeneity of the evaluated chemotherapy, the results have been inconsistent [22–25].

According to univariate and multivariate logistic regression analyses, we screened lymphovascular invasion, anemia, ER expression level, Ki67 expression level and NCT regimen as independent predictors, and then we constructed a nomogram to predict the probability of pCR in NCT patients. The AUC of the ROC curve was 0.758, indicating good predictive ability. We used a 5-fold cross validation model in a cohort of patients, and the final prediction accuracy was 76%. According to the Youden index and diagnostic odds ratios, we assigned an optimal cutoff value of 0.32. According to our results, patients with lymphovascular invasion, anemia (HB ≤ 120 g/L), ER positivity, and low Ki67 expression levels (≤60%) were most likely associated with a lower pCR rate from NCT.

Lymphovascular invasion is an independent prognostic parameter for poor outcome of invasive breast cancer and is the main prerequisite for metastasis [26, 27]. A previous study showed that lymphovascular invasion was significantly associated with chemoresistant breast cancer [19]. Another recent study reported that the absence of lymphovascular invasion in post-NCT specimens correlated with pathologic response [28]. Our research previously showed that lymphovascular invasion was related to a low pCR rate in NCT patients, which indicates that lymphovascular invasion may be an important molecular target in breast cancer. In addition, lymphovascular invasion was obtained before NCT treatment in our research, so we could know in advance whether the patient could reach pCR and then decide whether to administer neoadjuvant chemotherapy in these patients.

Previous studies have determined that anemia is an independent prognostic factor that adversely affects the survival of several cancer patients, including breast cancer patients [29, 30]. The impaired survival observed among cancer patients with anemia has been mainly due to a decrease in oxygen transport capacity, leading to tumor hypoxia [31]. Previous findings have suggested that anemia may result in worse treatment outcomes from breast cancer chemotherapy [32]. Another study [33] evaluated the influence of hemoglobin levels in 144 patients receiving chemotherapy and found that hemoglobin levels in patients who responded to treatment (tumor size reduced by more than 50%) were higher than those in patients who did not respond (*P* < 0.01). The hemoglobin concentration of 12.5 g/dl provided a significant cutoff value below which no reaction was likely to occur. This is the same as our research, in which a HB level ≤ 120 g/L had a lower pCR rate in patients treated with NCT.

Some prospective studies have shown that patients with HR-negative diseases can more often obtain pCR than those with HR-positive diseases [7, 34], which is the same as our results. Compared with patients with other subtypes, patients with estrogen receptor-positive tumors have a lower pCR rate from NCT. Most efficacy prediction models established in previous studies have also included the expression of HR. In addition, in previous studies [35], higher expression of PR was associated with a lower degree of response to adjuvant chemotherapy. In our research, the inclusion or exclusion of PR in multifactor analysis did not significantly affect the prediction accuracy of the model. Additionally, PR was often analyzed in breast cancer tumors but was rarely taken into account. Therefore, the final prediction model did not include PR status.

Tumor cell proliferation indexes, such as baseline Ki67, can be used to predict the efficacy of NCT in breast cancer. Previous studies have shown a significant correlation between gene expression markers related to cell proliferation or genetic grading and chemosensitivity in ER-positive and ER-negative breast cancer [36]. ER-positive breast cancer can be further divided into different subtypes, namely, luminal A and luminal B subtypes, and these two subtypes have different prognoses [37]. A transformation study based on randomized clinical trials also confirmed that the Ki67 index before neoadjuvant chemotherapy was not only a predictor of efficacy but also a prognostic factor of breast cancer [38]. This study confirmed the importance of proliferation-related markers from the perspective of treatment and re-emphasized that the cell proliferation index should be included on the basis of clinical decision-making regarding breast cancer. However, the optimal cut-off value of Ki-67 expression for predicting NCT response still needs further study.

It has been well established that pCR varies depending on the treatment regimen and breast cancer subtype. In our research, the pCR rates of HER2-positive and triple-negative tumors were significantly higher than those of luminal B tumors, and the use of TCbH had a higher pCR rate than the use of TEC for chemotherapy. A study [39] showed that when NCT was combined with trastuzumab, patients with HER-2 overexpression had a higher pCR rate. The use of TCbH has shown encouraging results in the neoadjuvant environment [40]. In HER2-positive breast cancer patients who received chemotherapy combined with trastuzumab, the increase in the pCR rate was directly related to an improvement in the survival rate and a reduction in disease recurrence and death risk [41–43]. As a result, trastuzumab has become an integral part of the global guidelines for breast cancer treatment [44, 45].

Based on clinical biological factors, our nomogram has good predictive ability for the early efficacy of NCT for breast cancer. Compared with those of previous similar studies, the advantages of this study are specifically reflected by the following aspects. First, in addition to traditional clinicopathological factors, this study attempted to incorporate important biological factors into the predictive model, such as anemia and lymphovascular invasion. Chemotherapy sensitivity was assessed as accurately as possible by comprehensively evaluating multiple factors related to the efficacy of NCT. Second, in this study, patients received NCT with TEC or TCbH protocols, which complied with the latest version of the NCCN guidelines; the tested information of the enrollment was complete, and the research results were highly applicable and reliable. Finally, the pCR rate of NCT can be known only by routine inspection before operation, so we can quickly estimate the probability of individual NCT benefits and help doctors more effectively make clinical decisions.

Nevertheless, several limitations are worth noting in our present study. First, our observations were limited to retrospective studies from a single center, and our nomogram was not validated in an external cohort. Second, this model could be further improved by expanding its scope of application, such as HER2-positive breast cancer patients using trastuzumab and pertuzumab in combination. Finally, the sample size was relatively small, and the predictive ability of the model needs to be further verified in large-sample studies.

## Conclusions

our nomogram confirmed that patients with lymphovascular invasion, anemia (HB ≤ 120 g/L), ER positivity, and low Ki67 expression levels (≤60%) were most likely to be associated with a lower pCR rate from NCT. With the nomogram, we can predict the pCR rate from NCT by quantitative indicators. For patients who are operable and have a predicted pCR probability of less than 0.32, we consider that there is limited benefit from NCT and that these patients can be directly treated with surgery.

## Supplementary information

Additional file 1: **Supplementary Table 1.** Model development of risk point. Abbreviations: NCT: Neoadjuvant chemotherapy; LVI: lymphovascular invasion. **Supplementary Table 2.** Predictive values and sensitivity, specificity of the predicted probability at different cutoff values. Abbreviations: PPV: positive predictive value; NPV: negative predictive value. **Supplementary Table 3.** The diagnostic odds ratio (DOR) of the nomogram at different cutoff values. **Supplementary Table 4.** Predictive values and sensitivity, specificity of the predicted probability at the optimal cutoff value. Abbreviations: PPV: positive predictive value; NPV: negative predictive value

## Data Availability

The datasets used during the current study available from the corresponding author on reasonable request.

## Abbreviations

pCR: Pathologic complete response
NCT: Neoadjuvant chemotherapy
ER: Estrogen receptor
PR: Progesterone receptor
HER-2: Human epidermal growth factor receptor 2
BCS: Breast-conserving surgery
EFS: Event-free survival
OS: Overall survival
TEC: Docetaxel + epirubicin + cyclophosphamide
TCbH: Docetaxel + carboplatin + trastuzumab
IDC: Invasive ductal carcinoma
IHC: Immunohistochemical
ROC: Receiver operating characteristic
AUC: Area under the curve
OR: Odds ratio
CI: Confidence interval
BMI: Body mass index
LVI: Lymphovascular invasion
