# Supplementary material for: Nomogram-derived prediction of pathologic complete response (pCR) in breast cancer patients treated with neoadjuvant chemotherapy (NCT)
Source: BMC Cancer. 2020 Nov 19;20:1120. doi: 10.1186/s12885-020-07621-7 (PMC7678042; doi:10.1186/s12885-020-07621-7)
Supplement: Supplementary file 1 — Supplementary Table 1. Model development of risk point. Abbreviations: NCT: Neoadjuvant chemotherapy; LVI: lymphovascular invasion. Supplementary Table 2. Predictive values and sensitivity, specificity of the predicted probability at different cutoff values. Abbreviations: PPV: positive predictive value; NPV: negative predictive value. Supplementary Table 3. The diagnostic odds ratio (DOR) of the nomogram at different cutoff values. Supplementary Table 4. Predictive values and sensitivity, specificity of the predicted probability at the optimal cutoff value. Abbreviations: PPV: positive predictive value; NPV: negative predictive value [file 12885_2020_7621_MOESM1_ESM.docx]

Supplementary table 1：Model development of risk point

| **Effect** |  | **Score** |
| --- | --- | --- |
| Hemoglobin（g/L） | >120 | 63 |
| Hemoglobin（g/L） | ≤120 | 0 |
| NCT regimens | TEC | 0 |
| NCT regimens | TCbH | 87 |
| Ki67 | >60 | 100 |
| Ki67 | ≤60 | 0 |
| LVI | Absent | 85 |
| LVI | Present | 0 |
| Estrogen receptor | Negative | 79 |
| Estrogen receptor | Positive | 0 |

| Total Points | Probability of pCR |
| --- | --- |
| 111 | 0.05 |
| 166 | 0.10 |
| 226 | 0.20 |
| 266 | 0.30 |
| 298 | 0.40 |
| 328 | 0.50 |
| 358 | 0.60 |
| 391 | 0.70 |
| 430 | 0.80 |

Abbreviations: NCT: Neoadjuvant chemotherapy; LVI: lymphovascular invasion

Supplementary table 2: Predictive values and sensitivity, specificity of the predicted probability at different cutoff values.

| **Predicted Probability** | **Sensitivity (%)** | **Specificity (%)** | **ppv(%)** | **npv(%)** | **index** |
| --- | --- | --- | --- | --- | --- |
| 0.100 | 93.3 | 34.2 | 34.7 | 93.2 | 27.5 |
| 0.108 | 93.3 | 35.0 | 35.0 | 93.3 | 28.3 |
| 0.117 | 93.3 | 39.2 | 36.5 | 94.0 | 32.5 |
| 0.126 | 93.3 | 40.8 | 37.2 | 94.2 | 34.2 |
| 0.204 | 91.1 | 42.5 | 37.3 | 92.7 | 33.6 |
| 0.208 | 82.2 | 53.3 | 39.8 | 88.9 | 35.6 |
| 0.223 | 80.0 | 57.5 | 41.4 | 88.5 | 37.5 |
| 0.238 | 73.3 | 65.8 | 44.6 | 86.8 | 39.2 |
| 0.254 | 66.7 | 68.3 | 44.1 | 84.5 | 35.0 |
| 0.262 | 66.7 | 69.2 | 44.8 | 84.7 | 35.8 |
| 0.296 | 62.2 | 73.3 | 46.7 | 83.8 | 35.6 |
| 0.320 | 62.2 | 77.5 | 50.9 | 84.5 | 39.7 |
| 0.456 | 60.0 | 78.3 | 50.9 | 83.9 | 38.3 |
| 0.499 | 33.3 | 90.8 | 57.7 | 78.4 | 24.2 |
| 0.505 | 20.0 | 98.3 | 81.8 | 76.6 | 18.3 |
| 0.526 | 20.0 | 99.2 | 90.0 | 76.8 | 19.2 |
| 0.765 | 20.0 | 100.0 | 100.0 | 76.9 | 20.0 |

Abbreviations: PPV: positive predictive value；NPV: negative predictive value

Supplementary table 3：The diagnostic odds ratio (DOR) of the nomogram at different cutoff values.

| **Predicted Probability** | **DOR (95% CI)** |
| --- | --- |
| ≥0.1 | 7.266(2.123-24.872) |
| ≥0.2 | 7.576(2.551-22.502) |
| ≥0.3 | 5.673(2.708-11.884) |
| ≥0.4 | 5.423(2.593-11.341) |
| ≥0.5 | 14.750(3.047-71.396) |


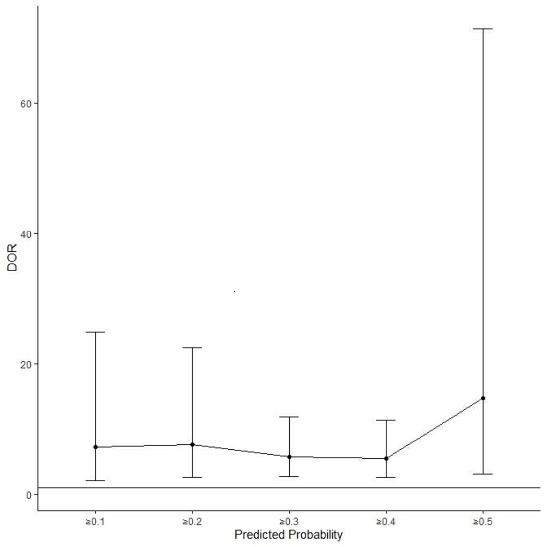


Supplementary table 4: Predictive values and sensitivity, specificity of the predicted probability at the optimal cutoff value.

|  | **The Optimal Cutoff** | **Sensitivity (%)** | **Specificity (%)** | **ppv(%)** | **npv(%)** |
| --- | --- | --- | --- | --- | --- |
|  | 0.32 | 62.2 | 77.5 | 50.9 | 84.5 |

Abbreviations: PPV: positive predictive value；NPV: negative predictive value
